# Transcription factor expression landscape in *Drosophila* embryonic cell lines

**DOI:** 10.1186/s12864-024-10241-1

**Published:** 2024-03-23

**Authors:** Robert A. Drewell, Daniel Klonaros, Jacqueline M. Dresch

**Affiliations:** Biology Department, Clark University, 950 Main Street, Worcester, MA 01610 USA

**Keywords:** *Drosophila*, Transcription factor, Embryo, Cell lines, Kc, S2

## Abstract

**Background:**

Transcription factor (TF) proteins are a key component of the gene regulatory networks that control cellular fates and function. TFs bind DNA regulatory elements in a sequence-specific manner and modulate target gene expression through combinatorial interactions with each other, cofactors, and chromatin-modifying proteins. Large-scale studies over the last two decades have helped shed light on the complex network of TFs that regulate development in *Drosophila melanogaster*.

**Results:**

Here, we present a detailed characterization of expression of all known and predicted *Drosophila* TFs in two well-established embryonic cell lines, Kc167 and S2 cells. Using deep coverage RNA sequencing approaches we investigate the transcriptional profile of all 707 TF coding genes in both cell types. Only 103 TFs have no detectable expression in either cell line and 493 TFs have a read count of 5 or greater in at least one of the cell lines. The 493 TFs belong to 54 different DNA-binding domain families, with significant enrichment of those in the zf-C2H2 family. We identified 123 differentially expressed genes, with 57 expressed at significantly higher levels in Kc167 cells than S2 cells, and 66 expressed at significantly lower levels in Kc167 cells than S2 cells. Network mapping reveals that many of these TFs are crucial components of regulatory networks involved in cell proliferation, cell–cell signaling pathways, and eye development.

**Conclusions:**

We produced a reference TF coding gene expression dataset in the extensively studied *Drosophila* Kc167 and S2 embryonic cell lines, and gained insight into the TF regulatory networks that control the activity of these cells.

**Supplementary Information:**

The online version contains supplementary material available at 10.1186/s12864-024-10241-1.

## Background

Cellular identities and functions depend on differential gene expression, which occurs primarily at the transcriptional level and is controlled by complex regulatory networks of transcription factors (TFs). Specifically, TF proteins bind DNA by interacting with specific sequences in regulatory elements to activate or repress transcription. Modulated through protein interactions and signaling pathways, TFs control the spatial and temporal transcriptional programs that ultimately specify cell fates and coordinate tissue and organ formation during development. Genomic technologies have enabled detailed annotation of regulatory networks in multiple biological contexts and have increased our understanding of regulatory connections. Several valuable large-scale studies over the last 20 years have helped elucidate the complex network of TFs that regulate development in *Drosophila melanogaster* [1–8].

Further comprehension of the control of gene expression in *Drosophila* requires the integration of a systematic analysis of the expression profile of TFs in defined cellular systems. Two of the most widely studied cell lines from *Drosophila* are Kc167 [9] and Schneider 2 [10] cells. Both of these cell lines were isolated from embryos and have been extensively used in wide-ranging studies of biological processes [11]. Kc167 (Kc) cells were derived from embryos at stage 13–15 (dorsal closure) [12] [13], while Schneider 2 (S2) cells originate from embryos at stage 16–17 (late embryonic) [10]. Although both cell lines display evidence of a hematopoietic origin, their respective global gene expression profiles are distinct [14, 15] and the patterns of TF coding gene expression in each cell type remains poorly characterized.

In this current work, we expand on these earlier studies by utilizing genome-wide RNA sequencing approaches to systematically characterize expression for all known or predicted *Drosophila* TFs in Kc and S2 cells. Deep read coverage enables us to compare the transcription profile for all 707 annotated TFs in the two cell lines in detail. The results shed light on some key shared features and differences between the two embryonic cell types and contribute to our understanding of the transcriptional landscape in the cell lines. Network analysis uncovers some important components of the regulatory environment in the cells and opens up the possibility of using these cell lines to further investigate critical TFs involved in the molecular control of gene regulatory networks in embryonic development.

## Methods

### Cell culture and RNA isolation

The Kc167 (Kc, RRID: CVCL_Z833) and S2-DRSC (S2, RRID: CVCL_Z992) cell lines used in this study were obtained from the *Drosophila* Genomics Resource Center (DGRC). Cells were thawed, passaged and frozen as previously described [15]. Cells were harvested at ~ 5 × 10^6^ cells/mL density from six replicate samples grown in 25cm^2^ canted neck culture flasks (Corning) and RNA isolated as previously described using a RNeasy kit following the manufacturer’s protocol (Qiagen) [15].

### RNA sequencing and read mapping

Library construction and sequencing were performed at the Beijing Genomics Institute. Briefly, 10ug of total RNA was enriched for poly(A)^+^ RNA by oligo(dT) selection and used to generate a cDNA library for sequencing, as previously described [15]. The libraries were sequenced on the Illumina nanoball (DNBSEQ) PE100 platform. Sequencing data was filtered to remove reads that contained adaptor sequences, reads whose N content was greater than 5%, and low-quality reads (quality score less than 15 for 20% or greater of the total bases in the given read). The generated clean read fastq files were aligned using Bowtie2 software to the *Drosophila melanogaster* genome (Release 6 plus ISO1 mitochondrial, RefSeq accession: GCF_000001215.4) and used to calculate quantitative RPKM, FPKM and TPM scores as previously described [15].

### Transcription factor gene expression analysis

A detailed computational pipeline enhanced with manual curation was employed by the Berkeley *Drosophila* Genome Project to identify a comprehensive list of 708 genes that encode a putative DNA-binding domain (DBD) in the *Drosophila melanogaster* genome [2]. Upon analysis of the complete list of 708 genes we discovered one duplication (gene symbols *mamo* and *CG11071*). We therefore removed *CG11071* and the total number of transcription factor (TF) genes considered in our study was 707 (Table S1). Of these 707 genes, 604 demonstrated detectable expression in either Kc or S2 cells, and 493 had read counts of 5 or greater in at least one of the cell lines (Table S2).

Classification of the individual TFs by DBD was performed using the GO TF level 2 annotation term in the *phyper* R package (https://stat.ethz.ch/R-manual/R-devel/library/stats/html/Hypergeometric.html), in combination with the *qvalue* Bioconductor package (https://bioconductor.org/packages/release/bioc/html/qvalue.html). GO enrichment analysis was initially performed in the *phyper* R package to calculate *p*-values. A *q*-value for each family of DBD TFs was obtained by multiple testing correction of the *p*-value, with a final *q*-value <  = 0.05 considered a significant enrichment. A TF enrichment ratio (Rich Ratio) for each DBD family was calculated by dividing the number of candidate genes identified with a specific DBD term, with the total number of genes in the genome annotated with the same DBD term.

Differentially expressed genes were defined as genes with a False Discovery Rate (FDR) equal to or less than 0.001 and fold change equal to or greater than 2. The R package *pheatmap* was used to perform hierarchical clustering analysis on the sets of differentially expressed genes, as previously described [15].

Dr. Tom (http://biosys.bgi.com) was used to evaluate the protein–protein interaction (PPI) network of the TF encoding genes. All the nodes in the PPI network were TF mRNAs. KEGG pathway enrichment analysis [16] was performed using the same methodology as the GO functional enrichment analysis described above. Pathways with a final *q*-value <  = 0.05 are defined as significantly enriched in differentially expressed genes. The KEGG pathways of the selected genes are ranked by the number of genes in the pathway and only the top 10 pathways with the largest number of genes are displayed.

### Data availability

The datasets supporting the results of this article are available at the NCBI Sequence Read Archive (SRA) under BioProject accession number PRJNA937779.

## Results and discussion

### Transcription factor expression landscape

In order to consider the expression of a complete set of DNA-binding transcription factors (TFs) in the *Drosophila* genome, we compiled a list containing 707 genes (Table S1). The list was based on detailed prior studies that utilized a combination of computational prediction from a DNA-binding domain (DBD) database [17] enhanced with manual annotation to identify all putative TF coding genes in the genome [2] (see Methods for details). As previously reported, we identified a total of 73 distinct DBD families represented in this comprehensive list of 707 genes [2].

**Fig. 1:**
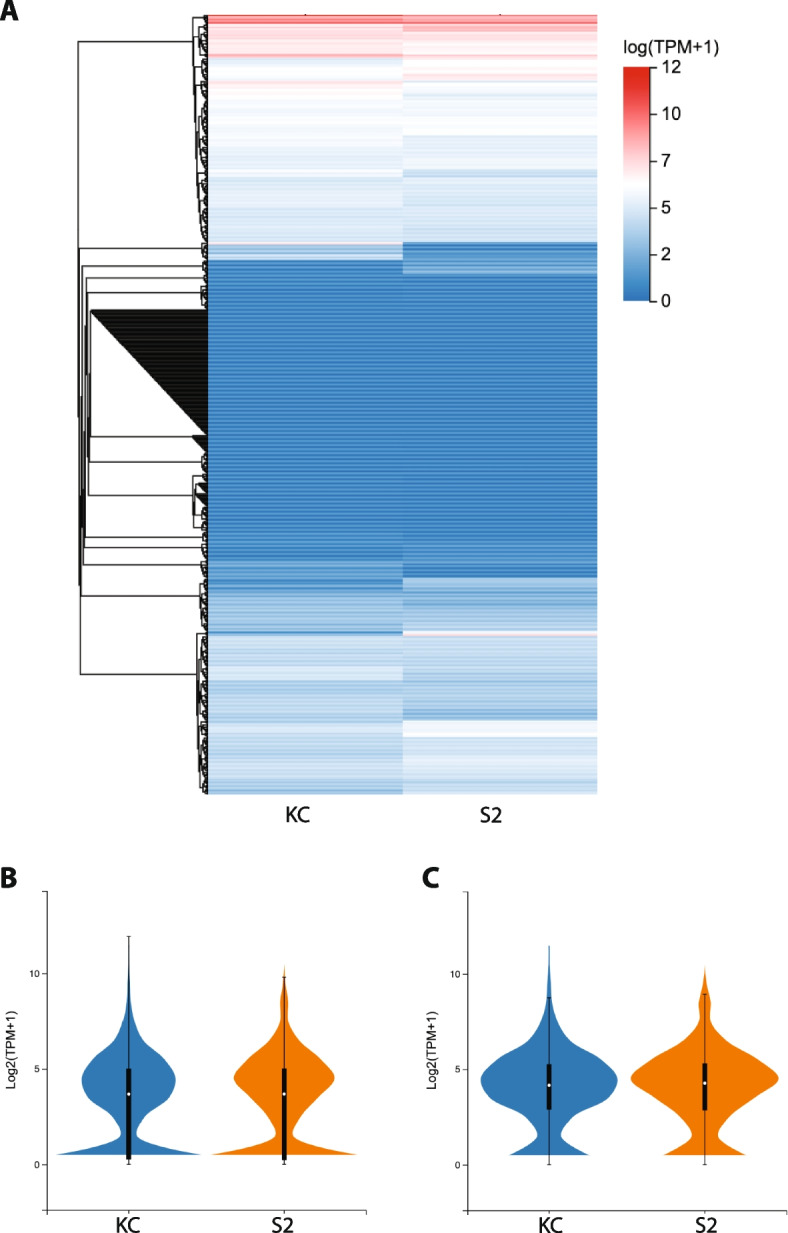
Expression profile of all 707 *Drosophila* transcription factor genes. **A** Heatmap indicates log_2_ expression level for all 707 annotated TF genes in the *Drosophila* genome in Kc and S2 cells. The expression level color key is shown to the right. **B** Violin plot indicating maximum, upper quartile, median, lower quartile and minimum TPM expression (log_2_ scale) for all 707 TF genes in Kc (dark blue) and S2 (orange) cells. The median value in Kc cells is 3.671 and in S2 cells is 3.676. **C** Violin plot indicating maximum, upper quartile, median, lower quartile and minimum TPM expression (log_2_ scale) for the 493 TF genes with a read counts of 5 or greater in at a least one cell type (as described in methods) in Kc (dark blue) and S2 (orange) cells. The median value in Kc cells is 4.155 and in S2 cells is 4.265

Gene expression of the 707 TF genes in both cell lines is exponentially distributed, varying from undetectable to 3,932 transcripts per million (TPM) in Kc cells and undetectable to 892 TPM in S2 cells, with a majority of genes expressed at the lower end of this range (Fig. 1a and Table S1). The median TPM expression value (log_2_ scale) in Kc cells is 3.671 and in S2 cells is 3.676 (Fig. 1b). Expression was detectable in at least one of the two cell line transcriptomes for 604 genes, which equals 85.43% of the 707 total annotated TFs in the genome. A group of 493 of the 707 genes (69.73%) had a read count of 5 or greater in at least one of the cell lines (Table S2), with median TPM expression values in Kc cells of 4.155 and in S2 cells of 4.265 for this group (Fig. 1c).

### Comparison to existing datasets

The overall expression profiles for the TF genes in both cell lines are largely consistent with earlier modENCODE studies [14]. Analysis across 25 different *Drosophila* cell lines, including Kc and S2, revealed that 228 of the 711 identified TFs with characterized DNA-binding domains were not expressed at all in any of the cell lines examined [14]. For the remaining 483 TFs, there was a wide range in levels of expression across the different cell types. We analyzed the expression of the 27 TFs shown to have the greatest variation in expression across the 25 cell lines analyzed in the modENCODE studies (Table S3). Seventeen of the 27 demonstrate at least a two-fold change in expression between the Kc and S2 cell types in our study (see Methods for full details), with many mirroring the reported differences between these cell lines in the earlier study [14]. Two TFs (*sug* and *ham*) have very high expression in both cell lines and two TFs (*noc* and *HLH4C*) have relatively low expression in both cell lines, in agreement with the prior studies (Table S3). In contrast, we found that the remaining six TFs (*twi*, *ac*, *bi*, *Dr*, *HGTX* and *hbn*) have no detectable expression in either cell type, representing a difference between our dataset and the modENCODE results.

We also analyzed the expression of 16 TF genes previously shown to have ubiquitous and high level expression in all 25 different *Drosophila* cell lines [14]. Reassuringly, all 16 of these genes demonstrated very high levels of expression in both Kc and S2 cells in our study, with no significant differences in the levels of expression between the two cell types (Table S4). As these TFs are expressed uniformly in all *Drosophila* cell lines, we propose that they may represent a signature for immortalization and would be the core set of candidate TFs to study the regulation of *Drosophila* cell replicative proliferation in future studies.

### 493 expressed TFs classified by DBD family

**Fig. 2:**
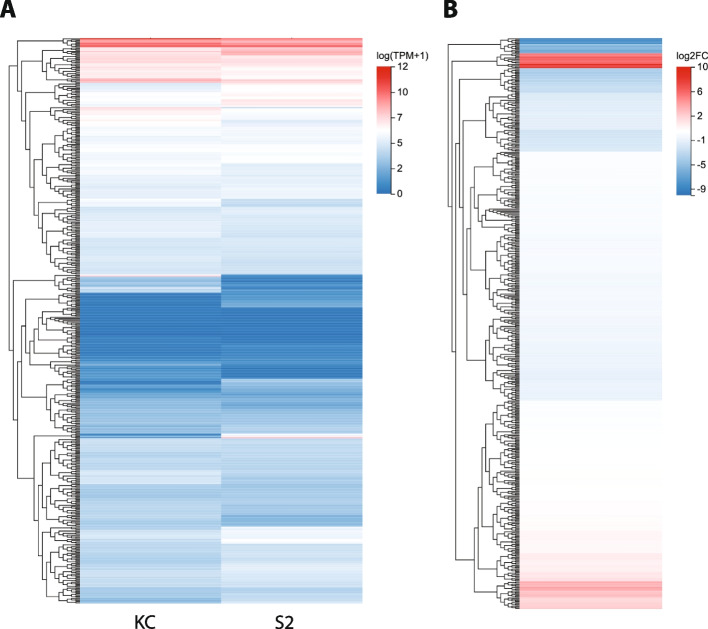
Expression profile of the 493 transcription factor genes with read counts of 5 or greater in Kc and/or S2 cells. **A** Heatmap indicates log_2_ expression level for the 493 TF genes in Kc and S2 cells. The expression level color key is shown to the right. **B** Heatmap indicates log_2_ fold change for the 493 TF genes in Kc and S2 cells. The fold change ratio (log_2_ Kc/S2) color key is shown to the right. Genes indicated in red are more highly expressed in Kc cells than S2 cells. Genes indicated in blue are more highly expressed in S2 cells than Kc cells.

**Fig. 3:**
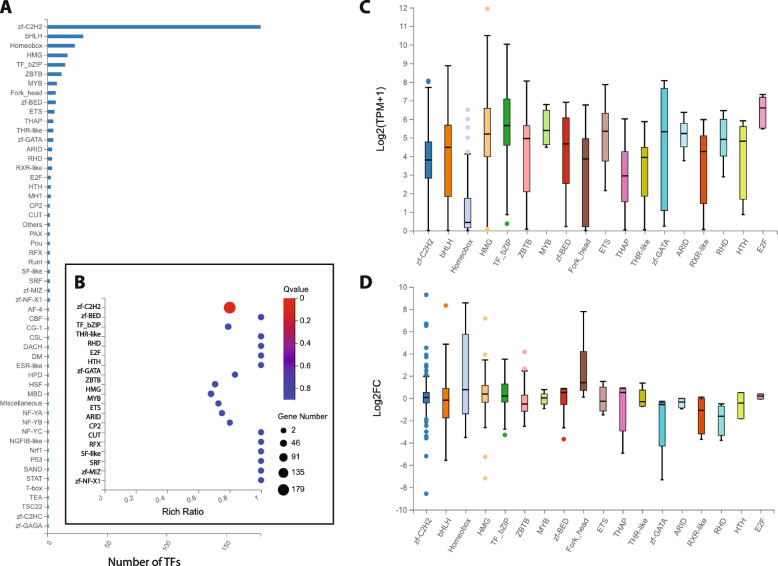
Classification of the 493 transcription factor genes by DNA-binding domain. **A** Histogram of the 493 TF genes annotated DNA-binding domain (DBD) sorted by frequency. **B** Enrichment analysis of the TF DBD families. The color key for the calculated *q*-value for each class is shown to the right and the size of the data point for each DBD family is representative of the total number of genes in that class. **C** Boxblot indicating maximum, upper quartile, median, lower quartile and minimum TPM expression (log_2_ scale) in Kc and S2 cells for TF genes in the 18 most frequent DBD families. Outlying data points are indicated with individual dots. **D** Boxblot indicating maximum, upper quartile, median, lower quartile and minimum fold change (log_2_ scale) between Kc and S2 cells for TF genes in the 18 most frequent DBD families. Positive values represent genes more highly expressed in Kc cells than S2 cells, while negative values represent genes more highly expressed in S2 cells than Kc cells. Outlying data points are indicated with individual dots

If we consider just the 493 TF coding genes with a read count of 5 or greater in at least one of the cell lines (Table S2), the majority are expressed at relatively low levels in both cell types (Fig. 2a) and consequently do not demonstrate a significant fold change in expression between the two cell types (Fig. 2b). However, a number of genes do show distinct expression profiles when compared between Kc and S2 cells (Fig. S1). The distribution of the 493 TFs by their annotated DBD is shown in Fig. 3a. Of the 73 DBD families present in the comprehensive list of 707 TFs, 54 (73.97%) different families are represented in the group of 493 TFs. Of those, 13 are present in five or more TFs, with the zinc finger zf-C2H2 family as the largest group with 179 members, followed by the bHLH (30 TFs) and homeobox (23 TFs) groups (Fig. 3a). Twenty-four distinct DBD families are present in only one TF each.

Analysis of the enrichment in the 493 TFs (see Methods for details) reveals 20 different DBD families with at least 2 different TFs and a Rich Ratio greater than 0.5 (Fig. 3b). Amongst those, only the zf-C2H2 family, with 179 of the 224 total TF genes in the genome, is significant (0.799 Rich Ratio, *q*-value = 8.88 × 10^–14^). Eleven of the DBD families demonstrate a Rich Ratio of 1, indicating that every member of that particular DBD family in the genome is present on the list of 493 TFs. However, none report a significant *q*-value score (Fig. 3b), likely due to the very low number of total annotated genes in these families (ranging from 2 to 7).

If we consider the overall expression level in both cell types of the 493 TFs classified by DBD family, the median TPM expression values (log_2_ scale) range from 0.43 to 6.61 (Fig. 3c). Notably, the members of the homeobox family have the lowest median expression level amongst the 18 most frequent DBD families, although a few outlying homeobox TF genes are expressed at higher levels (Fig. 3c). When the difference in expression between Kc and S2 cells is analyzed for the 18 most frequent DBD families, the median fold change (log_2_ scale) values all fall in the narrow range of -1.62 to 1.39 (Fig. 3d). However, within almost all 18 different DBD families there is extensive variation from the minimum to maximum fold change value and six different DBD families (zf-C2H2, bHLH, HMG, bZIP, ZBTB and zf-BED) contain individual TF genes with outlying fold change values (Fig. 3d).

### Differentially expressed TF genes

**Fig. 4:**
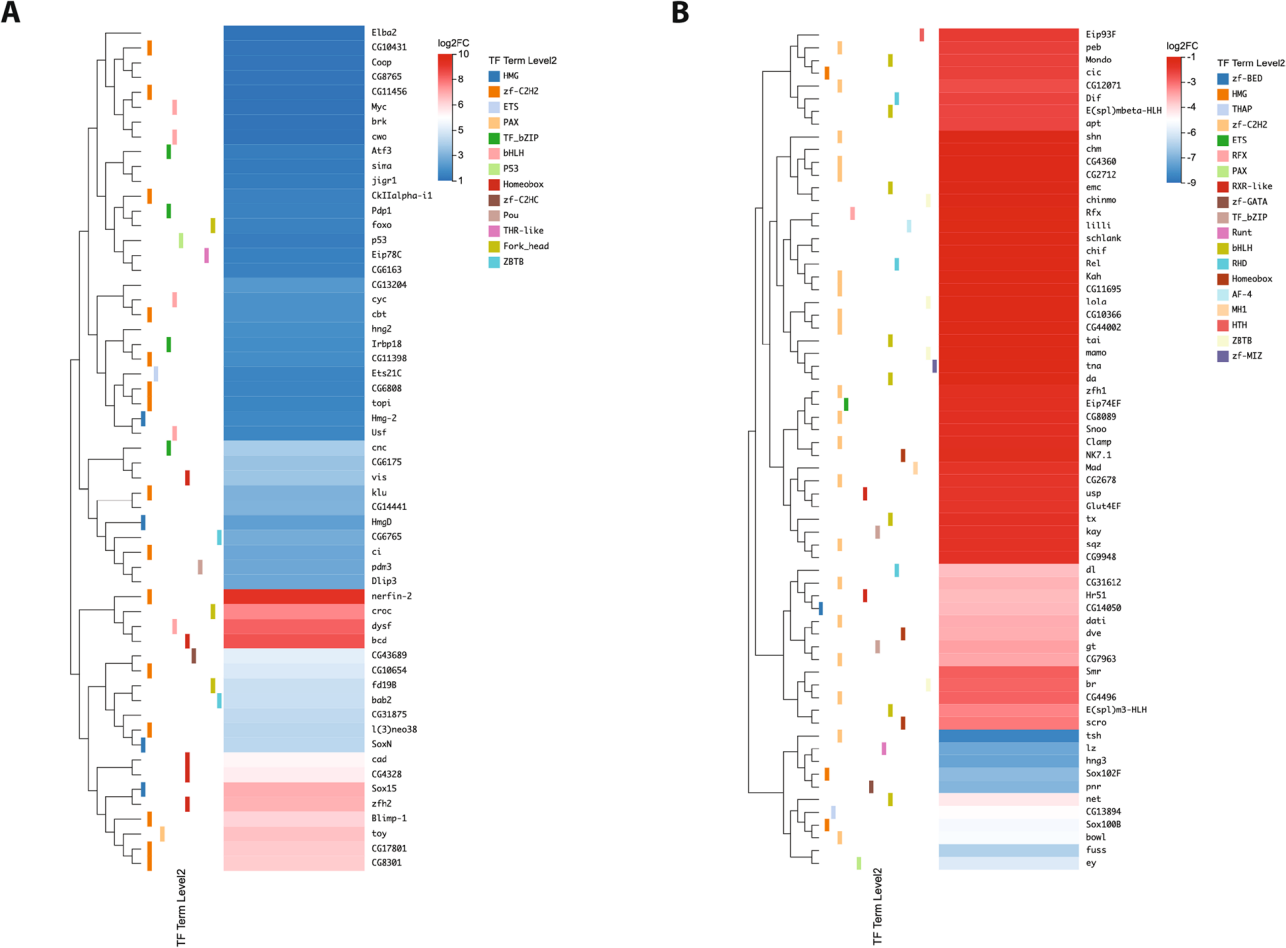
Difference heatmaps for DEGs. Heatmaps indicate log_2_ fold change for the 57 genes expressed at significantly higher levels in Kc cells when compared to S2 cells (Kc up) **(A)** and the 66 genes that are expressed at significantly higher levels in S2 cells than Kc cells (Kc down) **(B)**. The annotated functional GO TF level 2 classification term for each gene is indicated to the left of the heatmap with color key to the far right. The gene name and fold change ratio (log_2_ Kc/S2) color key is shown to the right

In order to further characterize the expression profile differences amongst the 493 TFs in Kc and S2 cells, we identified 123 differentially expressed genes (DEGs) with at least a two-fold change in expression between the two cell types (see Methods for full details). Of these DEGs, 57 are expressed at significantly higher levels in Kc cells than S2 cells (Kc up, Table 1) and 66 are expressed at significantly lower levels in Kc cells than S2 cells (Kc down, Table 2). Organizing the DEGs in expression heatmaps along with their annotated DBD, reveals that the 57 Kc up TFs are contained in 13 different DBD families (Fig. S2a), while the 66 Kc down TFs are in 19 different DBD families (Fig. S2b). A heatmap of the fold change in expression between the two cell types demonstrates that many, but certainly not all, of the TF genes show a relatively modest fold change (Fig. 4). Taken together with the overall expression profile for all 707 TFs, this indicates that the transcriptional landscape for the TF coding genes in the genome may be shared to a certain degree between the two cell lines.

**Table 1:** Upregulated DEGs ranked by log_2_ fold change

Gene Symbol	Gene ID	KC TPM	S2 TPM	KC Read Count	S2 Read Count	log2 (KC / S2)
nerfin-2	41235	7.99	0	269	0	9.29
bcd	40830	4.84	0	168	0	8.57
dysf	43174	176.21	0.54	8689	26	8.32
croc	40374	5.63	0.03	181	1	7.78
Sox15	36575	16.51	0.12	835	6	7.16
zfh2	43795	1.77	0.01	253	2	7.11
toy	43833	21.78	0.19	773	6	6.83
CG17801	42144	6.59	0.06	107	1	6.69
CG8301	41160	83.34	0.81	3559	35	6.65
Blimp-1	38638	1.16	0.02	71	1	6.49
CG4328	39405	0.78	0	21	0	5.93
cad	35341	1.36	0.03	46	1	5.74
CG43689	31353	0.34	0	32	0	4.75
CG10654	39428	1.18	0.06	26	1	4.52
fd19B	33010	2.8	0.15	36	2	4.19
bab2	44254	8.01	0.44	574	32	4.16
CG31875	318996	15.58	0.97	211	13	3.98
SoxN	44275	0.77	0.05	43	3	3.91
l(3)neo38	41423	5.54	0.37	327	17	3.90
cnc	42743	152.08	13.13	10551	1051	3.51
vis	36372	8.77	0.87	213.59	22.28	3.31
CG6175	39271	15.98	1.65	870	86	3.24
CG14441	31611	1.1	0.15	91	12	2.84
klu	39228	2.85	0.41	186	24	2.80
CG6765	38971	6.3	0.99	295.9	46.86	2.64
pdm3	35813	1.45	0.25	104	16	2.50
Dlip3	53579	9.4	1.66	186	33	2.48
ci	43767	1.69	0.3	116	22	2.46
HmgD	37481	3932.31	805.31	51211	10794	2.26
CG13204	36227	15.55	3.75	396	93	2.02
cyc	40162	58.44	15.56	1283	344	1.88
cbt	33224	267.85	72.71	7564	2049	1.85
hng2	41056	41.25	11.82	662	191	1.78
CG11398	31070	4.35	1.3	90	40	1.72
Irbp18	39243	91.21	27.43	682	204	1.71
Hmg-2	37407	47.21	15.15	851	275	1.61
Usf	31384	21.31	7.16	909	309	1.55
Ets21C	33229	39.01	13.51	1423	521	1.50
topi	41199	13.77	4.82	502	177	1.49
CG6808	41394	21.09	7.46	410	146	1.47
CkIIalpha-i1	39721	12.3	4.5	263	97	1.42
Pdp1	45588	70.72	26.36	4358	1625	1.40
foxo	41709	24.84	9.28	1276	486	1.39
CG6163	39274	9.17	3.48	204	79	1.37
Eip78C	40345	11.53	4.43	594	237	1.35
p53	2768677	46.45	18.29	937	383	1.32
Atf3	43867	21.86	8.9	2136	878	1.27
jigr1	43093	39.95	16.48	893	370	1.25
sima	43580	65.16	27.2	4843	2118	1.23
CG10431	35157	1.53	0.7	61	26	1.09
CG8765	40147	35.41	16.43	1506	704	1.08
Coop	35677	26.85	12.47	539	251	1.08
brk	31665	25	11.74	1103	522	1.06
cwo	44669	52.42	24.78	3354	1632	1.05
Myc	31310	72.31	34.36	6538	3448	1.05
CG11456	40309	10.65	5.08	215	103	1.04
Elba2	33442	6.32	3.07	127	62	1.02

The 57 genes expressed at significantly higher levels in Kc cells when compared to S2 cells (Kc up) are listed with the read count, TPM values and log_2_ expression ratio for the two cell types

**Table 2:** Downregulated DEGs ranked by log_2_ fold change

Gene Symbol	Gene ID	KC TPM	S2 TPM	KC Read Count	S2 Read Count	log2 (KC / S2)
tsh	35430	0.17	62.22	11	4129	-8.57
hng3	38090	0	2.76	0	44	-7.78
lz	31883	0.02	5.14	1	240	-7.68
pnr	44849	1.41	221.13	55	8652	-7.32
Sox102F	43844	0.08	11.03	3	506	-7.19
fuss	43835	0.06	4.84	2	194	-6.59
ey	43812	0.05	2.8	2	120	-5.80
Sox100B	45039	0.04	1.46	1	41	-5.27
bowl	33602	0.95	34.25	45	1639	-5.20
CG13894	38086	0.03	1.16	1	35	-4.94
net	45339	0.13	3.23	4	82	-4.55
dl	35047	6.44	87.07	253	3524	-3.78
Hr51	36702	0.04	0.49	1	14	-3.70
CG14050	31218	0.16	2.09	3	39	-3.67
CG31612	35427	0.8	9.55	38	448	-3.59
dve	37546	0.03	0.29	2	18	-3.52
dati	43789	0.11	1.27	5	58	-3.49
CG7963	41001	0.21	2.26	3	32	-3.40
gt	31227	0.29	2.84	7	69	-3.30
E(spl)m3-HLH	43156	0.52	3.87	9	68	-2.95
scro	3355151	0.99	6.81	26	170	-2.80
br	44505	9.3	52.6	649	4276	-2.53
CG4496	34000	0.69	3.79	22	116	-2.48
Smr	32225	13.02	68.81	2522	13390	-2.43
CG12071	43660	0.47	2.05	17	75	-2.14
apt	37734	9.14	36.4	330	1427	-2.02
E(spl)mbeta-HLH	43152	5.46	21.64	70	279	-2.01
Dif	35045	13.55	52.51	623	2311	-1.98
cic	53560	24.53	92.6	2829	10180	-1.94
Mondo	35402	25.88	97.62	1402	5326	-1.94
peb	31391	0.37	1.37	39	139	-1.91
Eip93F	44936	0.82	2.88	130	472	-1.84
usp	31165	19.12	62.07	665.53	2163.49	-1.73
Glut4EF	41217	5.13	16.62	361	1333	-1.72
CG2678	40937	7.49	24.16	192	616	-1.72
Mad	33529	14.82	47.07	553	1775	-1.69
kay	3772082	115.27	355.35	5393	16809	-1.65
sqz	42300	5.02	15.25	226	692	-1.63
CG9948	38757	11	33.02	117	375	-1.61
tx	43190	1.18	3.48	39	116	-1.59
CG8089	36679	2.42	6.78	66	186	-1.51
Snoo	5740414	5.59	15.65	479.53	1432.64	-1.51
Eip74EF	39962	3.44	9.5	249	676	-1.49
zfh1	43650	93.03	257.02	8460	23596	-1.49
Clamp	35445	40.18	107.82	1471	3995	-1.45
NK7.1	41747	17.77	45.97	747	1958	-1.40
lilli	33496	11.44	27.21	1209	2779	-1.28
Rfx	41266	2.07	4.92	128	321	-1.27
Rel	41087	28.28	66.47	1251	2981	-1.26
Kah	38072	1.95	4.57	76	179	-1.25
CG11695	32106	13.56	31.61	321	755	-1.25
schlank	50392	128.45	298.29	4025	9345	-1.24
chif	34974	12.1	28.01	1106.71	2555.57	-1.24
lola	44548	117.88	264.61	5228.28	11234.91	-1.19
CG44002	33535	4	8.95	68	153	-1.19
CG10366	35258	9.6	21.46	270	608	-1.19
tai	34242	17.93	39.46	2037	4507	-1.17
tna	39217	12.86	27.98	807	1855	-1.15
da	34413	40.23	87.24	1759	3851	-1.14
mamo	32353	14.06	30.37	1733	4468	-1.14
chm	43928	9.01	19.25	342	698	-1.12
CG4360	42413	60.22	128.6	2933	6305	-1.12
CG2712	31267	5.56	11.84	136	292	-1.12
chinmo	33343	28.82	60.55	3935	7583	-1.10
emc	38091	47.06	98.52	1315	2773	-1.09
shn	36171	15.37	31.31	2010	4139	-1.05

The 66 genes expressed at significantly higher levels in S2 cells when compared to Kc cells (Kc down) are listed with the read count, TPM values and log_2_ expression ratio for the two cell types

**Fig. 5:**
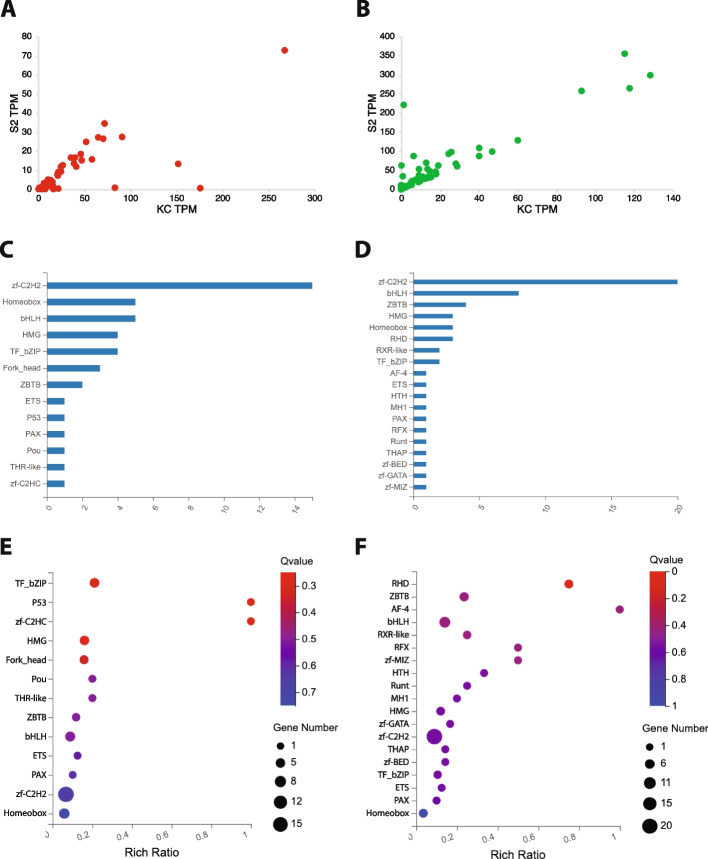
Classification of DEGs by DNA-binding domain. Scatterplots comparing the TPM value for the 56 genes (the *HmgD* gene, ID #37841 is not shown due to very high expression in Kc cells) expressed at significantly higher levels in Kc cells when compared to S2 cells (Kc up) **(A)** and the 66 genes that are expressed at significantly higher levels in S2 cells than Kc cells (Kc down) **(B)**. Histograms of the frequency of annotated DNA-binding domains (DBDs) in the 57 Kc up genes **(C)** and the 66 Kc down genes **(D)**. Enrichment analysis of the TF DBD classes in the 57 Kc up genes **(E)** and the 66 Kc down genes **(F)**. The color key for the calculated *q*-value for each class is shown to the right and the size of the data point for each DBD class is representative of the total number of genes in that class

Despite these overall similarities, if we analyze the TPM values of the DEGs in the two cell types there are genes with disparate expression profiles, including a number of genes with high level expression in one cell type and very low TPM values in the other cell type (Fig. 5a, b). The distribution of the 57 Kc up TFs and the 66 Kc down TFs by their respective annotated DBD is shown in Fig. 5c, d. Thirteen different DBD families are represented in the group of 57 TFs, with the zinc finger zf-C2H2 family as the largest group with 15 members, followed by the homeobox and bHLH groups (5 TFs each). Six DBD families are present in only one TF each (Fig. 5c). In the group of 66 Kc down TFs, 19 different DBD families are represented, with the zinc finger zf-C2H2 family as the largest group with 20 members, followed by the bHLH group (8 TFs) and ZBTB (4 TFs). Eleven DBD families are present in only one TF each (Fig. 5d). Analysis of the enrichment of the DEGs classified by their DBD reveals a range of values, including two DBD families (P53 and zf-C2HC) amongst the 57 Kc up TFs (Fig. 5e) and one DBD family (AF-4) amongst the 66 Kc down TFs (Fig. 5f) with a Rich Ratio of 1. However, it should be noted that all three of those DBD families are represented by a single TF encoding gene. None of the DBD families in either group of DEGs report a significant *q*-value score (Fig. 5e, f).

**Fig. 6:**
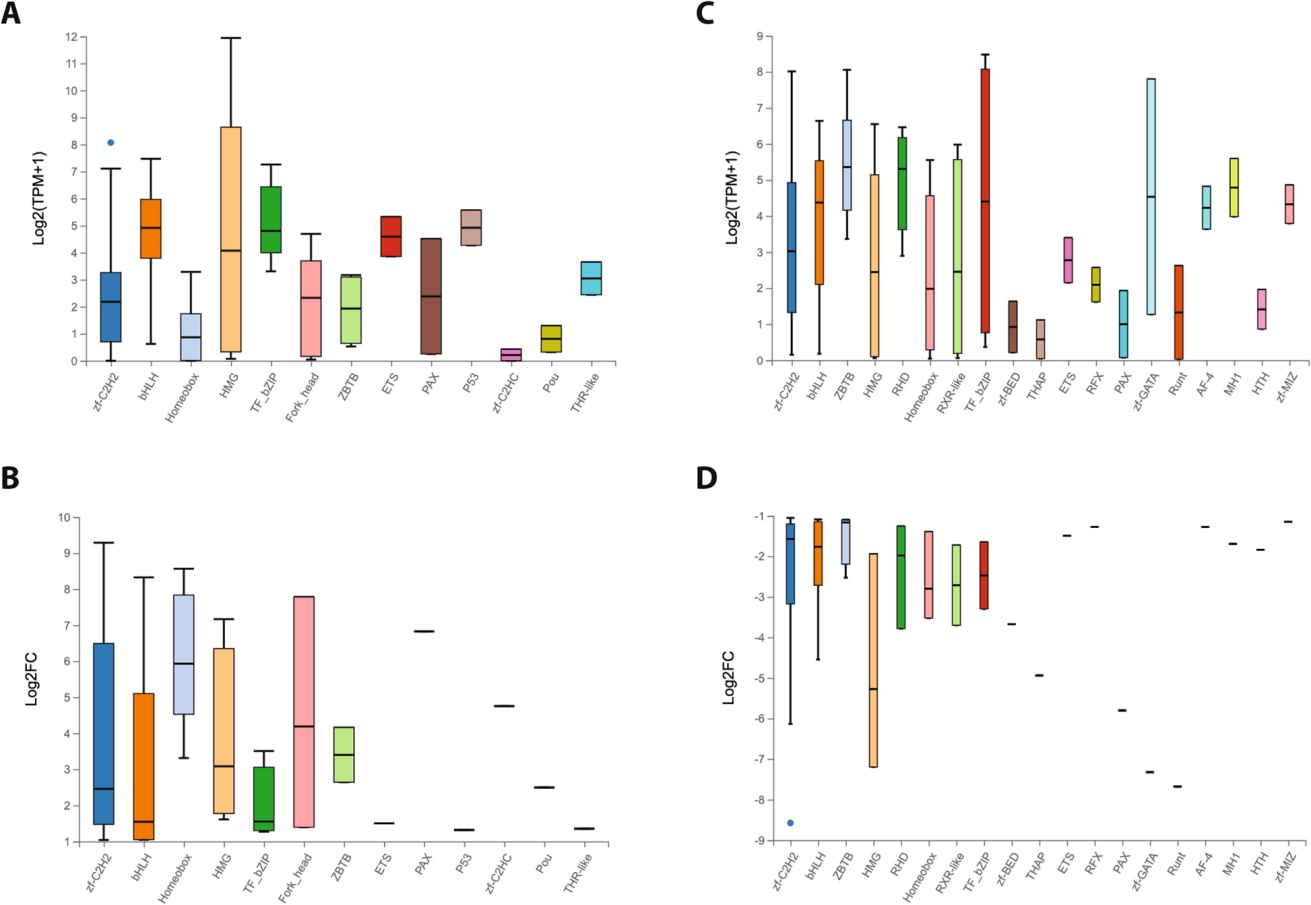
DEG DNA-binding domain groups expression and difference profile. **A** Boxblot indicating maximum, upper quartile, median, lower quartile and minimum TPM expression (log_2_ scale) in Kc and S2 cells for the 57 Kc up TF genes organized by DNA-binding domain (DBD) classes. Outlying data points are indicated with individual dots. **B** Boxblot indicating maximum, upper quartile, median, lower quartile and minimum fold change (log_2_ scale) between Kc and S2 cells for the 57 Kc up TF organized by DBD classes. **C** Boxblot indicating maximum, upper quartile, median, lower quartile and minimum TPM expression (log_2_ scale) in Kc and S2 cells for the 66 Kc down TF genes organized by DNA-binding domain (DBD) classes. Outlying data points are indicated with individual dots. **D** Boxblot indicating maximum, upper quartile, median, lower quartile and minimum fold change (log_2_ scale) between Kc and S2 cells for the 66 Kc down TF genes organized by DBD classes. Outlying data points are indicated with individual dots

If we consider the overall expression level in both cell types of the DEGs classified by DBD family, the median TPM expression values (log_2_ scale) range from 0.21 to 4.92 in the 13 families representing the 57 Kc up TFs (Fig. 6a), and 0.58 to 5.36 in the 19 families representing the 66 Kc down TFs (Fig. 6c). When the difference in expression between Kc and S2 cells for the DBD families is analyzed, the median fold change (log_2_ scale) values for the Kc up group fall in the range of 1.32 to 6.83 (Fig. 6b), with the Kc down group in the range of -1.15 to -7.68 (Fig. 6d). For all of the 14 DBD families across the two DEG groups with more than one TF member, there is extensive variation from the minimum to maximum fold change value (Fig. 6b, d).

### Hematopoietic cell identity

To investigate the hematopoietic identity of the two cell lines we analyzed expression of known TF gene markers within this lineage (Table S5). In *Drosophila*, three distinct types of hemocytes originate from a common precursor stem-cell like population: plasmatocytes, crystal cells and lamellocytes [18]. Previous studies have indicated that Kc cells have a plasmatocyte identity and that S2 cells combine some properties of plasmatocyte and crystal cells [14, 15]. In our RNA-seq data, we detect very high levels of the GATA-like prohemocyte marker *serpent* (*srp*) in Kc and S2 cells (Table S5). In addition, both cell types express the Friend of GATA homolog *u-shaped (ush)*, which regulates the population size of crystal cells and can act as an inhibitor of crystal cell differentiation [19], and *scalloped* (*sd*), which promotes crystal cell specification in a *Notch/Serrate*-dependent manner [20]. This discrepancy could in part be due to the detection sensitivity in our current study, although the level of expression we detect for these two genes is very high. The differentiating prohemocyte markers *glial cells missing* (*gcm*) and *gcm2* are not expressed in either cell type (Table S5), indicating that neither cell line has the molecular profile of an intermediate prohemocyte [20]. S2 cells do express a detectable level of the Runt domain TF *lozenge* (*lz*) and *pebbled* (*pb*), both of which are associated with *Notch*-dependent crystal cell differentiation and not with plasmatocytes [20–22]. Expression of these two crystal cell markers is very low in Kc cells (Table S5). Intriguingly, *klumpfuss* (*klu*), which has been shown to inhibit pre-destined crystal cells from becoming plasmatocytes [20], is expressed at a higher level in Kc cells than S2 cells.

We also compared our data with single-cell transcriptome analysis in *Drosophila* embryos. Examining expression in embryos at the onset of gastrulation (stage 6) using the *Drosophila* Virtual Expression eXplorer (DVEX) package [23] reveals that *srp* and *ush*, both of which are expressed at a high level in both Kc and S2 cells with no significant differences in the levels of expression between the two cell types (Table S5), are co-expressed in previously identified t-distributed stochastic neighbor embedding (t-SNE) cell clusters [23]. These two genes are predominantly co-expressed in t-SNE cluster 6 (Fig. S3), which is one of the 11 spatially identified clusters grouped by transcriptome similarity in the DVEX project, and corresponds to the dorsal epidermis of the embryo [23]. The crystal cell marker *pb*, which we detect at a higher level in S2 cells than Kc cells, is also predominantly expressed in t-SNE cluster 6 (Fig. S3d). It should be noted that expression of *srp*, *ush*, and *pb* is also detectable in sub-populations of cells within additional t-SNE clusters (Fig. S3) and expression of *sd* is very widespread in stage 6 embryos.

In summary, our new data confirm that both Kc and S2 cells display TF expression profiles indicative of a hematopoietic origin, but that the lines are distinct from each other. The S2 cells appear to have a more prominent crystal cell identity, based on *lz and pb* expression, while the Kc cells exhibit many of the hallmarks of plasmatocyte identity. However, both cell types display a certain level of transcriptional plasticity not seen in any particular hemocyte cell type found in the embryo, suggesting that further analysis, including single cell transcriptomics on the cell lines, will be required to clarify the precise molecular identity of the cells.

### Protein–protein interaction network analysis

**Fig. 7:**
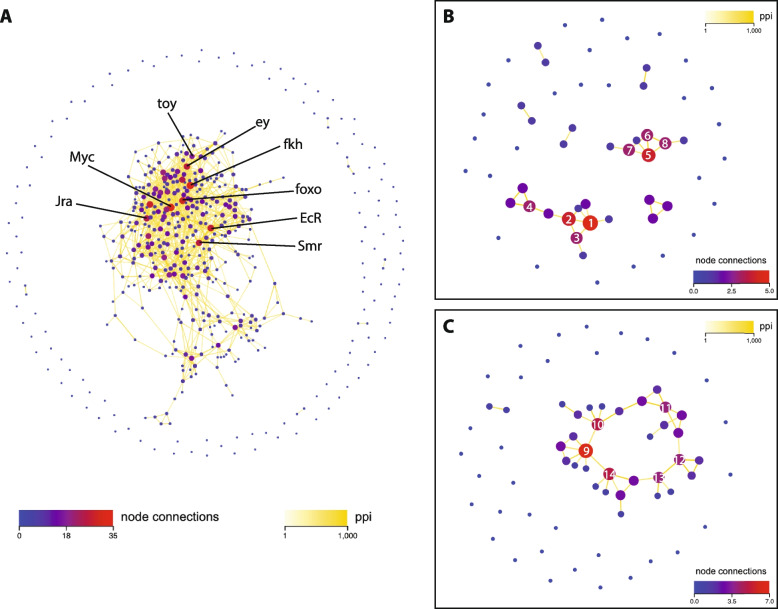
Protein–protein interaction network analyses. **A** PPI network map for all 493 TF genes. Individual genes with high connectivity are labeled. **B** Interactions between the 57 TF genes that are expressed at significantly higher levels in Kc cells than S2 cells (Kc up). Individual genes are indicated by number; 1 = *Myc*, 2 = *foxo*, 3 = *p53*, 4 = *cyc*, 5 = *cad*, 6 = *toy*, 7 = *Sox15*, 8 = *SoxN*. **C** Interactions between the 66 TF genes that are expressed at significantly higher levels in S2 cells than Kc cells (Kc down). Individual genes are indicated by number; 9 = *ey*, 10 = *Mad*, 11 = *usp*, 12 = *Rel*, 13 = *kay*, 14 = *da*. Circles indicate individual TF coding genes (nodes) and are color coded according to the number of total connections from each node as indicated in the *node connections* color code key. The line color connecting the nodes indicates the relative strength of the calculated PPI value as shown in the *ppi* color key

To investigate the potential biological roles of the TF genes that are expressed in the two embryonic cell types, we performed an analysis of the molecular networks in the cells. The protein–protein interaction (PPI) network map of the 493 TFs demonstrates considerable interaction for the majority of TF genes (Fig. 7a). Of the 493 total genes, only 128 (25.96%) have zero node connections. Of the 365 genes (74.04%) with connections, the total number of connections for each gene ranges from 1 to 35 (Fig. 7a). Amongst the genes with a high level of connectivity (≥ 17 connections), some intriguing network components are revealed (Fig. 7a). The *Myc* gene (35 connections) encodes a TF homologous to the well-characterized vertebrate *Myc* proto-oncogene and has a critical role in cell growth and proliferation [24, 25]. Given that the two *Drosophila* cell lines are immortalized it is not surprising to find this gene expressed in both Kc (72.31 TPM) and S2 (34.36 TPM) cells. Likewise, the *Jra* (*Jun-related antigen*) gene (28 connections) encodes for a homolog of the mammalian *Jun* proto-oncogene TF [26, 27]. The *EcR* (*Ecydysone receptor*) gene (27 connections) encodes for a protein that forms the nuclear ecdysone receptor heterodimer with the protein product of *usp* (*ultraspiracle*) [28]. The heterodimer TF modulates expression of hundreds of downstream genes through sequence-specific binding to ecdysone response elements (ECRES) in the regulatory regions of target genes [29]. In addition, the functional activity of the heterodimer is known to be controlled by an array of co-activators and co-repressors, many of which, including the co-repressor *Smr* (*Smrter*, *SMRT-related ecdysone receptor interacting protein*) [30] (25 connections), are captured on the PPI network map (Fig. 7a). The *foxo* gene (29 connections) encodes for a TF involved in the regulation of the insulin signaling pathway and plays a key role in regulation of the cell cycle, modulating cell growth and proliferation [31]. Two less predictable components of the PPI network are represented by the *fkh* (*forkhead*) gene (34 connections), which encodes for a pleiotropic TF most frequently studied for its role in salivary gland formation [32], and the *ey* (*eyeless*) (26 connections) and *toy* (*twin of eyeless*) (17 connections) gene pair, that encode for critical TFs involved in eye development as part of the retinal determination gene network [33].

If we analyze just the 57 Kc up TFs, 28 (49.12%) have zero PPI connections, while 29 (50.88%) have between 1 and 5 connections (Fig. 7b). A key cluster in the network includes the *Myc*, *foxo*, *p53*, and *cyc* (*cycle*) genes, indicating that the TFs encoded by these genes play a critical role in regulating the cell cycle and proliferation in the Kc cell line. An interesting additional cluster is identified in the network containing the *cad* (*caudal*), *toy*, *Sox15* (*Sox box protein 15*), and *SoxN* (*Sox Neuro*) genes (Fig. 7b). The CAUDAL TF has a well-studied role in early embryonic patterning [34], but is also known to play a role in innate immune homeostasis [35], which may fit with the previously characterized hemocyte-like identity of the Kc cells [15]. The potential role of the TFs encoded by the *toy*, *Sox15* and *SoxN* genes in Kc cells is more enigmatic. All three of these TFs have characterized activity in the formation of the central nervous system [36–38], but are also known to be important for cell proliferation [39–41]. Unraveling the precise functional role of the *toy* and *Sox15* encoded TFs in future studies will be particularly appealing, given that they are expressed at significantly higher levels in Kc cells (*toy* 21.78 TPM and *Sox15* 16.51 TPM) than in S2 cells (*toy* 0.19 TPM and *Sox15* 0.12 TPM) (Table 1).

Amongst the 66 Kc down TFs, 33 (50%) have zero PPI connections, while 33 (50%) have between 1 and 7 connections (Fig. 7c). Thirty-one of the 33 genes with connectivity form a single large cluster in the network, which includes the *ey*, *Mad* (*Mothers against dpp*), *usp*, *Rel* (*Relish*), *kay (kayak*), and *da* (*daughterless*) genes (Fig. 7c). The TFs encoded by all these genes are implicated in regulating cell cycle and proliferation, although not necessarily in the same organ, tissue or system. The *ey*, *kay*, and *Mad* gene products have characterized roles in eye development [42–44], the *Rel* gene is important for the immune deficiency pathway [45], and the TF encoded by *da* participates in transcriptional regulation of a wide variety processes, including oogenesis, neurogenesis, myogenesis and cell proliferation [46] and is critical for sex determination and dosage compensation by controlling the feminizing switch gene *Sxl* (*Sex lethal*) [47]. It should be noted that for many of the genes in the single large network cluster, there is relatively high expression in both S2 and Kc cells (Table 2), suggesting that the biological activity of the encoded TFs may be functionally important in both cell lines. This potential shared role in the two cell lines makes these genes key candidates for further characterization in future studies.

### KEGG pathway analysis

**Fig. 8:**
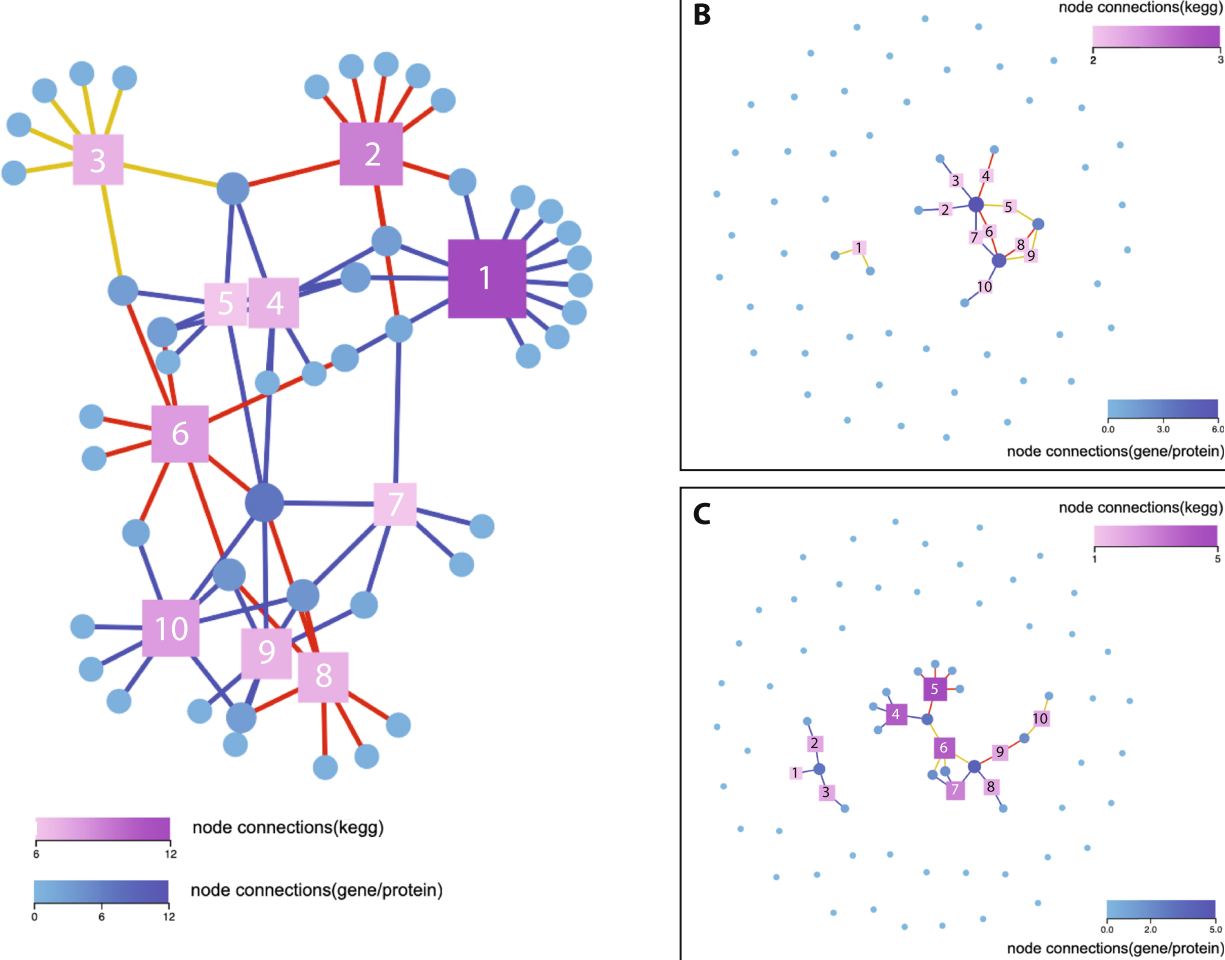
KEGG pathway analyses. **A** The 493 genes in the TF network were clustered using KEGG pathway analysis. KEGG pathway nodes are indicated by number; 1 = MAPK signaling pathway – fly, 2 = Apoptosis, 3 = Longevity regulating pathway, 4 = MAPK signaling pathway, 5 = PI3K-Akt signaling pathway, 6 = Cellular senescence, 7 = Hippo signaling pathway – fly, 8 = Signaling pathways regulating pluripotency of stem cells, 9 = Hippo signaling pathway, 10 = TGF-beta signaling pathway. **B** KEGG pathway analysis of the 57 TF genes that are expressed at significantly higher levels in Kc cells than S2 cells (Kc up). KEGG pathway nodes are indicated by number; 1 = Circadian rhythm, 2 = TGF-beta signaling pathway, 3 = Hippo signaling pathway – fly, 4 = Signaling pathways regulating pluripotency of stem cells, 5 = Thyroid hormone signaling pathway, 6 = Cellular senescence, 7 = PI3K-Akt signaling pathway, 8 = Mitophagy, 9 = Longevity regulating pathway, 10 = FoxO signaling pathway. **C** KEGG pathway analysis of the 66 genes that are expressed at significantly higher levels in S2 cells than Kc cells (Kc down). KEGG pathway nodes are indicated by number; 1 = Hippo signaling pathway, 2 = TGF-beta signaling pathway, 3 = Hippo signaling pathway – fly, 4 = MAPK signaling pathway – fly, 5 = Apoptosis, 6 = Toll and lmd signaling pathway, 7 = Ras signaling pathway, 8 = Sphingolipid signaling pathway, 9 = Cellular senescence, 10 = Thyroid hormone signaling pathway. Squares indicate defined KEGG pathways (nodes) and circles indicate individual TF coding genes. The KEGG pathway nodes are color ranked by the number of genes connected in the pathway, with only the top 10 pathways with the largest number of genes displayed in each panel. The different line colors connecting nodes to genes represent KEGG classification of the pathway; cellular processes (red), environmental information processing (blue) or organic systems (yellow)

KEGG pathway mapping was performed to investigate the link between our TF expression data and the underlying biological pathways in the two embryonic cell types. Analysis of the top 10 pathways for the group of 493 TFs reveals that each node (representing a particular biological pathway) has at least 6 connections to individual TF encoding genes, with the node of highest degree having 12 connections (Fig. 8a). Seven of the 10 nodes represent signaling pathways (including *Signaling pathways regulating pluripotency of stem cells*) with extensive interconnectivity between the nodes, indicating that regulation of cell–cell signaling is important in Kc and S2 cells. This result reinforces observations from earlier studies which showed that many parallel signaling pathways are active and that the signaling landscape is extensively shared between the two cell types [14, 15]. Amongst the KEGG signaling pathways identified, the fly MAPK (Mitogen activated protein kinase) pathway is well represented with 12 connections (Fig. 8a). Given that the protein encoded by the *Myc* gene is known to be one of the key TFs for the downstream regulation controlled by the MAPK signaling cascade [25], the KEGG pathway data ties in with the fact that expression of *Myc* was detected in both cell types. The three identified KEGG nodes not annotated as cell signaling pathways are; *Apoptosis*, *Longevity regulating pathway*, and *Cellular senescence*. Given the high proliferative activity of the two cell lines, the appearance of these pathways is perhaps not surprising.

The KEGG mapping for the top 10 pathways amongst the 57 Kc up TFs uncovers a large cluster of 9 interconnected nodes, the majority of which represent cell–cell signaling pathways (Fig. 8b). The separate single node represents the *Circadian rhythm* pathway and supports the earlier observation that the *cyc* gene, which encodes a TF known to activate transcription of key downstream circadian clock genes [48], is expressed (58.44 TPM) in Kc cells (Table 1). A similar profile for the KEGG mapping is observed for the top 10 pathways amongst the 66 Kc down TFs, with two clusters of interconnected nodes present (Fig. 8c). Eight of the ten nodes are explicitly annotated as cell–cell signaling pathways, with the other two nodes (*Apoptosis* and *Cellular senescence*) demonstrating connectivity to cell signaling mechanisms through shared genes (Fig. 8c).

In an effort to explore the connection between KEGG identified pathways and the functional requirements of specific genes within those pathways, we examined the JAK/STAT pathway, as a proof of principle, in detail. Our previous studies have revealed that the core *domeless* (*dome)* receptor and *unpaired 2* (*upd2*) and *unpaired 3* (*upd3*) ligands in the JAK/STAT pathway are expressed in both Kc and S2 cells [15]. A detailed study from the Sheffield RNAi Screening Facility (SRSF) utilized a second-generation, computationally optimized dsRNA library to perform a genome-wide RNAi screen in Kc cells to identify 42 regulators of JAK/STAT signaling [49]. Of the 42 genes identified, nine encode for TFs (Table S6). Amongst those nine genes, seven are ranked in the top 41 by overall expression level in Kc cells and the other two have very high levels of expression (ranked 159 and 162 overall). Furthermore, all nine TF genes also demonstrated very high levels of expression in S2 cells in our study, with six of the nine genes showing no significant differences in the levels of expression between the two cell types (Table S6). Taken together, the data indicate that the TF components of the JAK/STAT pathway identified by RNAi are likely functionally critical in Kc and S2 cells, as evidenced by their uniform very high levels of expression. Overall, the confirmed widespread expression of many of the TF protein components in the cell–cell signaling network, and their presence in many different KEGG mapped signaling pathways in Kc and S2 cells, confirms that the cell lines will remain a valuable tool to study *Drosophila* cell–cell interactions in future studies.

## Conclusions

Our comprehensive analysis of TF expression from the *Drosophila* genome in the commonly utilized Kc and S2 embryonic cell lines has revealed a complex landscape, in which the majority of the TF coding genes are actively expressed, although at varying levels. Of the 707 annotated TF genes, 493 are expressed at a level of 5 or greater TPM in at least one of the cell types and 123 are expressed at significantly different levels between the two cell lines. The protein–protein interaction network displays the collective influences of these TFs on cellular function and highlights a potentially important role for TFs involved in cell proliferation, cell–cell signaling pathways, and, surprisingly, eye development. KEGG pathway analysis further elucidates the role of these active TFs in signaling and cell cycle regulatory pathways. Understanding whether the differential expression of the 123 TFs is chiefly due to a difference in biological function between the two cell lines despite both possessing hematopoietic origins, a difference in neutral accumulated mutations in these two immortalized cell types, a difference in the molecular control of the route to immortality, or a combination of these possibilities, will require further study. This dataset sheds light on the TF milieu at play in these two cell lines and will serve as a resource for future gene regulatory studies that make use of either of these embryonic cell lines.

### Supplementary Information

**Supplementary Material 1. **

**Supplementary Material 2. **

**Supplementary Material 3. **

**Supplementary Material 4. **

**Supplementary Material 5. **

**Supplementary Material 6. **

**Supplementary Material 7. **

**Supplementary Material 8. **

**Supplementary Material 9. **

## Data Availability

The datasets supporting the results of this article are available at the NCBI Sequence Read Archive (SRA) under BioProject accession number PRJNA937779.

## Abbreviations

Kc: Kc167
S2: Schneider 2
TF: Transcription Factor
DNBSEQ: Illumina nanoball PE100 sequencing
FDR: False Discovery Rate
DBD: DNA-binding domain
GO: Gene Ontology
DEG: Differentially Expressed Gene
TPM: Transcripts Per Million
PPI: Protein–Protein Interaction

## References

[1] Rhee DY (2014). Transcription factor networks in Drosophila melanogaster. Cell Rep.

[2] Hammonds AS (2013). Spatial expression of transcription factors in Drosophila embryonic organ development. Genome Biol.

[3] Graveley BR (2011). The developmental transcriptome of Drosophila melanogaster. Nature.

[4] Giot L (2003). A protein interaction map of Drosophila melanogaster. Science.

[5] Stanyon CA (2004). A Drosophila protein-interaction map centered on cell-cycle regulators. Genome Biol.

[6] Guruharsha KG (2011). A protein complex network of Drosophila melanogaster. Cell.

[7] Roy S (2010). Identification of functional elements and regulatory circuits by Drosophila modENCODE. Science.

[8] Nègre N (2011). A cis-regulatory map of the Drosophila genome. Nature.

[9] Echalier G, Ohanessian A (1969). Isolation, in tissue culture, of Drosophila melangaster cell lines. C R Acad Hebd Seances Acad Sci D.

[10] Schneider I (1972). Cell lines derived from late embryonic stages of Drosophila melanogaster. J Embryol Exp Morphol.

[11] Luhur A, Klueg KM, Zelhof AC (2019). Generating and working with Drosophila cell cultures: Current challenges and opportunities. Wiley Interdiscip Rev Dev Biol.

[12] Andres AJ, Cherbas P (1992). Tissue-specific ecdysone responses: regulation of the Drosophila genes Eip28/29 and Eip40 during larval development. Development.

[13] Cherbas P (1988). 26-[125I]iodoponasterone A is a potent ecdysone and a sensitive radioligand for ecdysone receptors. Proc Natl Acad Sci U S A.

[14] Cherbas L (2011). The transcriptional diversity of 25 Drosophila cell lines. Genome Res.

[15] Klonaros D, Dresch JM, Drewell RA (2023). *Transcriptome profile in Drosophila Kc and S2 embryonic cell lines*. G3 (Bethesda).

[16] Kanehisa M (2008). KEGG for linking genomes to life and the environment. Nucleic Acids Res.

[17] Kummerfeld SK, Teichmann SA (2006). DBD: a transcription factor prediction database. Nucleic Acids Res.

[18] Lebestky T (2000). Specification of Drosophila hematopoietic lineage by conserved transcription factors. Science.

[19] Fossett N (2001). The Friend of GATA proteins U-shaped, FOG-1, and FOG-2 function as negative regulators of blood, heart, and eye development in Drosophila. Proc Natl Acad Sci U S A.

[20] Koranteng F (2020). The Role of Lozenge in Drosophila Hematopoiesis. Mol Cells.

[21] Jacques C (2009). A novel role of the glial fate determinant glial cells missing in hematopoiesis. Int J Dev Biol.

[22] Yu S, Luo F, Jin LH (2018). The Drosophila lymph gland is an ideal model for studying hematopoiesis. Dev Comp Immunol.

[23] Karaiskos N (2017). The Drosophila embryo at single-cell transcriptome resolution. Science.

[24] Gallant P (1996). Myc and Max homologs in Drosophila. Science.

[25] Bellosta P, Gallant P (2010). Myc Function in Drosophila. Genes Cancer.

[26] Perkins KK (1990). The Drosophila Fos-related AP-1 protein is a developmentally regulated transcription factor. Genes Dev.

[27] Zhang K (1990). Drosophila homolog of the mammalian jun oncogene is expressed during embryonic development and activates transcription in mammalian cells. Proc Natl Acad Sci U S A.

[28] Koelle MR (1991). The Drosophila EcR gene encodes an ecdysone receptor, a new member of the steroid receptor superfamily. Cell.

[29] King-Jones K, Thummel CS (2005). Nuclear receptors–a perspective from Drosophila. Nat Rev Genet.

[30] Tsai CC (1999). SMRTER, a Drosophila nuclear receptor coregulator, reveals that EcR-mediated repression is critical for development. Mol Cell.

[31] Jünger MA (2003). The Drosophila forkhead transcription factor FOXO mediates the reduction in cell number associated with reduced insulin signaling. J Biol.

[32] Weigel D (1989). The homeotic gene fork head encodes a nuclear protein and is expressed in the terminal regions of the Drosophila embryo. Cell.

[33] Czerny T (1999). twin of eyeless, a second Pax-6 gene of Drosophila, acts upstream of eyeless in the control of eye development. Mol Cell.

[34] Mlodzik M, Fjose A, Gehring WJ (1985). Isolation of caudal, a Drosophila homeo box-containing gene with maternal expression, whose transcripts form a concentration gradient at the pre-blastoderm stage. EMBO J.

[35] Ryu JH (2008). Innate immune homeostasis by the homeobox gene caudal and commensal-gut mutualism in Drosophila. Science.

[36] Kammermeier L (2001). Differential expression and function of the Drosophila Pax6 genes eyeless and twin of eyeless in embryonic central nervous system development. Mech Dev.

[37] Miller SW (2009). Complex interplay of three transcription factors in controlling the tormogen differentiation program of Drosophila mechanoreceptors. Dev Biol.

[38] Savare J, Bonneaud N, Girard F (2005). SUMO represses transcriptional activity of the Drosophila SoxNeuro and human Sox3 central nervous system-specific transcription factors. Mol Biol Cell.

[39] Dichtel-Danjoy ML, Caldeira J, Casares F (2009). SoxF is part of a novel negative-feedback loop in the wingless pathway that controls proliferation in the Drosophila wing disc. Development.

[40] Bahrampour S (2017). Neural Lineage Progression Controlled by a Temporal Proliferation Program. Dev Cell.

[41] Blanco J (2010). Genetic interactions of eyes absent, twin of eyeless and orthodenticle regulate sine oculis expression during ocellar development in Drosophila. Dev Biol.

[42] Clements J (2009). Mutational analysis of the eyeless gene and phenotypic rescue reveal that an intact Eyeless protein is necessary for normal eye and brain development in Drosophila. Dev Biol.

[43] Cerrato A (2006). Genetic interactions between Drosophila melanogaster menin and Jun/Fos. Dev Biol.

[44] Wiersdorff V (1996). Mad acts downstream of Dpp receptors, revealing a differential requirement for dpp signaling in initiation and propagation of morphogenesis in the Drosophila eye. Development.

[45] Han ZS, Ip YT (1999). Interaction and specificity of Rel-related proteins in regulating Drosophila immunity gene expression. J Biol Chem.

[46] Smith JE, Cummings CA, Cronmiller C (2002). Daughterless coordinates somatic cell proliferation, differentiation and germline cyst survival during follicle formation in Drosophila. Development.

[47] Kan L (2017). The m^6^A pathway facilitates sex determination in Drosophila. Nat Commun.

[48] Hung HC (2007). Circadian transcription depends on limiting amounts of the transcription co-activator nejire/CBP. J Biol Chem.

[49] Fisher KH (2012). Advances in genome-wide RNAi cellular screens: a case study using the Drosophila JAK/STAT pathway. BMC Genomics.

