# Supplementary material for: Transcription factor expression landscape in Drosophila embryonic cell lines
Source: BMC Genomics. 2024 Mar 23;25:307. doi: 10.1186/s12864-024-10241-1 (PMC10960990; doi:10.1186/s12864-024-10241-1)

**Figure S1. Scatterplot comparing the TPM values of the 493 TF genes with read counts of 5 or greater in Kc and/or S2 cells.**


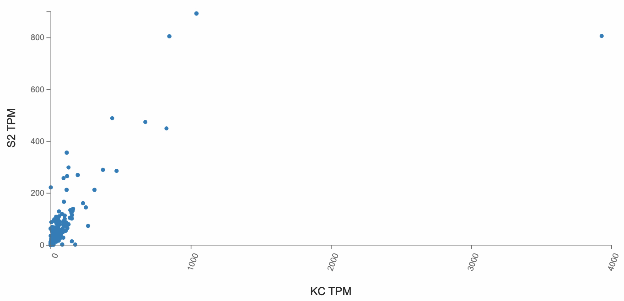

Supplement: Supplementary file 7 — Supplementary Material 7. [file 12864_2024_10241_MOESM7_ESM.docx]
