# Supplementary material for: Transcription factor expression landscape in Drosophila embryonic cell lines
Source: BMC Genomics. 2024 Mar 23;25:307. doi: 10.1186/s12864-024-10241-1 (PMC10960990; doi:10.1186/s12864-024-10241-1)
Supplement: Supplementary file 8 — Supplementary Material 8. [file 12864_2024_10241_MOESM8_ESM.docx]

**Figure S2. Differentially expressed TF genes heatmap.** The heatmaps indicate log_10_ expression level for the 57 TF genes that are expressed at significantly higher levels in Kc cells than S2 cells (Kc up) **(A)** and the 66 genes that are expressed at significantly higher levels in S2 cells than Kc cells (Kc down) **(B)**. The annotated functional GO TF level 2 classification term for each gene is indicated to the left of the heatmap with color key to the far right. The gene name and change ratio (log_2_ Kc/S2) color key is shown to the right.
