# Supplementary material for: Transcription factor expression landscape in Drosophila embryonic cell lines
Source: BMC Genomics. 2024 Mar 23;25:307. doi: 10.1186/s12864-024-10241-1 (PMC10960990; doi:10.1186/s12864-024-10241-1)
Supplement: Supplementary file 9 — Supplementary Material 9. [file 12864_2024_10241_MOESM9_ESM.docx]

**Figure S3. *Drosophil*a Virtual Expression eXplorer (DVEX) single-cell sequencing cluster identity. (A)** Two-dimensional t-SNE representation shows the eleven major clusters in stage 6 embryos grouped by transcriptome similarity. Expression of hemocyte marker TF genes, *serpent* (*srp*) **(B)**, *u-shaped* (*ush*) **(C)** and *pebbled* (*peb*) **(D),** in t-SNE clustered cells are shown. All data generated from DVEX package: <https://shiny.mdc-berlin.de/DVEX/>
